# Iron Chelation Therapy in Myelodysplastic Syndromes

**DOI:** 10.1155/2010/756289

**Published:** 2010-06-20

**Authors:** Emanuela Messa, Daniela Cilloni, Giuseppe Saglio

**Affiliations:** Division of Hematology and Internal Medicine, Department of Clinical and Biological Sciences of the University of Turin, Regione Gonzole 10, 10043 Orbassano (To), Italy

## Abstract

Myelodysplastic syndromes (MDS) are a heterogeneous disorder of the hematopoietic stem cells, frequently characterized by anemia and transfusion dependency. In low-risk patients, transfusion dependency can be long lasting, leading to iron overload. Iron chelation therapy may be a therapeutic option for these patients, especially since the approval of oral iron chelators, which are easier to use and better accepted by the patients. The usefulness of iron chelation in MDS patients is still under debate, mainly because of the lack of solid prospective clinical trials that should take place in the future. This review aims to summarize what is currently known about the incidence and clinical consequences of iron overload in MDS patients and the state-of the-art of iron chelation therapy in this setting. We also give an overview of clinical guidelines for chelation in MDS published to date and some perspectives for the future.

## 1. Introduction

Myelodysplastic syndromes (MDS) are clonal disorders of the hematopoietic stem cell and are mainly characterized by bone marrow blasts up to 20%, one or more peripheral cytopenias and bone marrow dysplasia [1]. The prognosis can be variable, with survival ranging from a few months to many years, and depends on the three features evaluated by the International Prognostic Scoring System (IPSS) [2]: cytogenetic abnormalities, percentage of blasts in the bone marrow, and number of peripheral cytopenias. Among so wide a spectrum of clinical features, clinicians must choose from different therapeutic options (reviewed in [3]), that vary from supportive care or growth factor administration to chemotherapy or bone marrow transplantation in younger and higher risk cases. New therapeutic options are now available; some of them, addressed to patients with specific cytogenetic features, such as lenalidomide for patients with 5q-. Other promising medications are the hypomethylating agents, which may improve survival in higher risk subjects [3].

Lower-risk MDS patients often become transfusion-dependent during the course of the disease, which can be quite long, and this could contribute to increased cardiac morbidity and mortality. It is well known that transfusion dependency is an important prognostic factor in MDS and portends worse prognosis [4]. Similarly, high ferritin level in refractory anemia, but not in refractory cytopenia without anemia, is a negative prognostic factor for survival [5]. However, it should be kept in mind that more aggressive disease is frequently associated with a high transfusion rate, so significant transfusion dependency often becomes a surrogate marker of aggressive disease.

Blood transfusion therapy may lead to organ toxicity due to the formation of nontransferrin bound iron (NTBI) and resulting oxidative stress, as is well documented in transfusion-dependent congenital anemias [6]. In low-risk MDS patients with longer life expectancy, preventing damage due to iron overload is an important concern. In fact, several authors underline the importance of iron chelation as a prognostic variable for improving survival [7, 8]. The data, however, are mainly derived from retrospective studies and need to be confirmed in prospective trials. Recently, Sanz et al. reported that iron overload (serum ferritin over 1000 ng/mL) has a negative prognostic impact on leukemia-free survival [9], suggesting that reactive oxygen species (ROS), increased in iron overload, may be responsible for DNA damage and disease progression in multiply transfused patients. Furthermore, other therapeutic indications have to be considered for iron chelation in MDS patients: pretransplant high ferritin level has a negative prognostic significance for survival in acute myeloid leukemia (AML) and MDS patients undergoing bone marrow transplantation [10, 11]; so many reports underline the usefulness of iron chelation for higher risk candidates for allografting. Moreover, Pullarkat et al. [12] recently have proposed that iron chelation may lower infection risk, delay leukemic transformation, and improve hematologic parameters in patients with higher IPSS scores who are not at this point generally considered to be candidates for chelation according to multiple guidelines [13–21]. This paper briefly summarizes what it is currently known about iron overload and iron chelation in MDS.

## 2. Iron Overload in MDS

Chronic transfusion therapy is the main cause of iron overload in MDS patients. It is well known from patients affected by congenital anemias that multiple transfusion leads to the formation of NTBI, which includes the labile iron pool (LIP) [6, 22]. This is defined as the chelatable iron, able to reach tissues and cells, and is thought to be responsible for tissue damage, fibrosis and organ failure, mainly affecting the liver, heart and pancreas [6]. Several reports describe organ damage possibly due to multiple transfusions in MDS patients [23, 24]. However, comorbidities often exist, making it difficult to discern how much organ damage is due to transfusion therapy as opposed to age-related comorbidities. Iron overload may have many clinical consequences in MDS patients, which are briefly summarized below.

## 3. Iron Overload and Impact on Survival and Leukemic Evolution

Several retrospective studies suggest an important contribution of transfusion dependency in shortening survival in MDS patients [25]. Malcovati et al. [26] reported in a series of 467 MDS patients a high incidence of cardiac failure (accounting for 51% of non leukemic deaths) in transfusion-dependent MDS patients. It is not yet clear how significant is the contribution of iron overload from transfusions, and how much is due to a higher disease severity in transfusion-dependent patients. However, a reduced survival rate has been observed in refractory anemia (RA) and refractory anemia with ringed sideroblasts (RARS) patients but not in refractory cytopenia with multilineage dysplasia (RCMD) patients [5]. More recently, the same group highlighted the importance of transfusion dependency for prognosis whether we consider leukemia-free or overall survival [27, 28]. Similarly, Cermak et al. [29] reported a statistically significant negative impact on survival of transfusi on dependency in MDS with erythroid dysplasia without excess blasts. The importance of iron overload on morbidity and mortality has been also shown by Takatoku et al. [30]. In this retrospective study which included 292 patients affected by MDS, myelofibrosis and aplastic anemia, 97% of deaths from cardiac and hepatic failure were observed in patients with a serum ferritin level over 1000 g/L. More recently, Sanz et al. analyzed the impact of iron overload in a multicenter study of the Spanish group including 2994 patients [9]. A multivariate analysis performed on 902 cases with complete data available demonstrated a strong association between not only high serum ferritin level and reduced survival (*P* < .0001), but also leukemia-free survival (*P* < .0001) [7]. This is the first study evaluating the correlation between iron overload and progression to leukemia, possibly suggesting a negative influence of reactive oxygen species in inducing increased genomic instability in hematopoietic progenitors, as has been demonstrated by in vitro and in vivo studies [31–33]. 

However, a strong and well-documented relation between shorter survival and iron overload is still lacking [34]. We need prospective studies able to correlate more precisely iron overload parameters such as NTBI measurement and appropriate magnetic imaging of the liver and heart to survival and leukemic progression.

## 4. Cardiac Iron and Transfusion Rate

Iron deposition in myocardial tissue is one of the most concerning potential complications in MDS patients receiving chronic transfusion therapy. It is well known that secondary hemocromatosis in thalassemia patients and in other congenital anemias leads to cardiac iron deposition and heart failure [35]. Many studies in recent years addressing this point in transfused MDS patients have been published. In this clinical setting we must consider that there is a shorter transfusional history compared with congenital anemias, and that other factors besides iron deposition could be involved in increased cardiac failure in transfused patients, such as anemia and MDS severity. Cardiac iron accumulation in MDS can be variable and there are many studies reporting conflicting results in this regard. In an autopsy series of transfusion-dependent patients, Buja and Roberts [36] found a direct correlation between the number of red cell transfusions and the amount of iron deposition. More recently, studies based on MRI T2* imaging suggest that cardiac iron accumulation is not a frequent feature of MDS patients [37] and does not correlate with serum ferritin levels or hepatic iron overload [38, 39] but only with the size of the chelatable iron pool [40].

## 5. Iron Overload and Risk of Infection

There are numerous observations suggesting an increased risk of infection in iron loaded patients, particularly those affected by acute leukemia or allogenic stem cell transplantation (SCT) recipients [41–43]. In these conditions, there is a coexistence of many factors able to enhance infectious susceptibility, such as neutrophil dysfunction, severe cytopenias and an increase in labile plasma iron (LPI) due either to a high transfusion rate or to myelosuppressive therapies. However, even if iron chelation can be useful in this setting in preventing bacterial and fungal infections, it is important to correctly choose the appropriate chelation agent: it has been suggested that deferoxamine can worsen fungal infections by acting as a siderophore [44]. In contrast, it has been demonstrated that deferasirox can be effective against mucormicosis infection in vivo [45]. So, as suggested recently by Pullarkat, iron chelators could have a possible new role in the future, lowering infection risks in MDS, expecially in high risk patients with more severe neutropenia [12].

## 6. Iron Overload in Transplantation

In recent years, different studies highlighted iron overload as a negative prognostic indicator in patients undergoing stem cell transplantation (SCT). In a cohort of 590 patients who underwent myeloablative SCT, Armand et al. [10] found a strong negative association between high serum ferritin level and overall and disease-free survival. This association was seen in patients affected by acute myeloid leukemia or myelodysplastic syndrome. The five-year overall survival decreased from 54% for patients with serum ferritin under 231 ng/mL to 27% for those with serum ferritin higher than 2034 ng/mL. Additionally, in MDS patients a serum ferritin higher than 2034 ng/mL was associated with a significantly increased transplant-related mortality (hazard ratio =   3,2), no correlation was found between serum ferritin level and relapse rate, however. Similar results have also been published by Pullarkat et al., who analyzed 190 patients undergoing SCT: in a multivariate analysis high pretransplant serum ferritin (above 1000 ng/ml) strongly correlated with an increased risk of death (hazard ratio 2,28, *P* = .004), day 100 mortality (odds ratio in generalized linear models of 3,82, *P* = .013), bloodstream infection (odds ratio = 3,11) and acute graft versus host disease (odds ratio = 1,99) [11]. The 190 patients were followed for a mean of 209 days: 27 died in the first 100 days and 29 after this cut off. The results of Cox proportional hazards model showed a higher risk of death in high-ferritin group (*P* = .004). More recently, Mahindra et al. studied 222 patients who underwent myeloablative allogeneic bone marrow transplantation: pretransplantation serum ferritin above 1910 *μ*g/l was strongly associated with lower overall and relapse-free survival (*P* = .003 for both the variables) and with lower incidence of chronic and acute graft versus host disease (*P* = .019 and 0,10, resp.). No differences were observed in relapse mortality and incidence of hepatic veno-occlusive disease between patients stratified according the serum ferritin value of 1910 *μ*g/l [46]. A higher morbidity rate in iron loaded transplant recipients has been shown by Au et al. [47]: high iron levels measured by T2* MRI mostly in the liver, pancreas and pituitary gland were associated with abnormal pancreas and pituitary function in 40%–70% of patients studied.

During the SCT procedure and follow up, serum ferritin and NTBI levels are markedly increased by several mechanisms (reviewed in [48]), increasing the risk of infections, mucositis, chronic liver disease, sinusoidal obstruction syndrome and idiopathic pneumonia. Therefore, reducing iron burden after SCT may be helpful in lowering the risk of infection [49] and organ toxicity, and can be achieved by phlebotomy in patients with a normal hemoglobin. Prospective clinical trials to evaluate the effectiveness and safety of iron chelators in this setting are ongoing [48].

## 7. Iron Chelation in MDS

### 7.1. Iron Chelators Commercially Available

Currently, three iron chelators are commercially available: deferoxamine (DFO), deferasirox (DFR) and deferiprone (DFP). DFO was introduced in the 1970s and had a profound impact on the survival of thalassemia patients [50]. In such patients the drug reduced organ dysfunctions and mortality, restoring a life expectancy similar to normal individuals with a survival rate strictly correlating to days of deferoxiamine infusions [51]. Due to its short half life, DFO is administered by subcutaneous infusion 5 to 7 nights/week, though it may be difficult to use in MDS patients due to thrombocytopenia. More recently, two oral agents have been introduced: the three-times daily agent deferiprone and the once-daily deferasirox [52–54]. DFP is effective in reducing hepatic and cardiac iron content in thalassemia patients, but its clinical use is partially limited by the risk of occurrence of agranulocytosis. This concerning adverse effect is rare, but potentially harmful in thalassemia and also in MDS, where pancytopenia is a common clinical feature. The latter agent, deferasirox, is administered once daily due to a long half life, and has been recently released on the market for the treatment of secondary iron overload in transfusion-dependent anemias. Several clinical studies evaluating the efficacy and safety of deferasirox in many transfusion-dependent congenital anemias have been published [55–58]. Moreover, its usefulness has also been tested in a cohort of MDS patients, yielding good data on efficacy and safety in this older population [59, 60]. Its side effects are generally mild, consisting mainly of nausea, diarrhea and a self-limiting serum creatinine increase, thus making this agent possibly the most suitable for chelation therapy in the MDS population [61]. We should also keep in mind the recent guidance from FDA regarding the use of deferasirox in MDS patients where a greater risk for adverse events such as kidney failure, gastrointestinal hemorrhage and deaths is reported for myelodysplastic patients compared to others without this condition (http:// www.fda.gov/Drugs/DrugSafety/PostmarketDrugSafetyInformationforPatientsandProviders/DrugSafetyInfor- mationforHealthcareProfessionals/ucm183651.htm).

### 7.2. Impact of Iron Chelation on Survival in MDS

Two retrospective studies reported a positive impact of iron chelation on the survival of MDS patients: Leitch et al. analyzed a population of 178 MDS patients from the University of British Columbia database, revealing a positive association between iron chelation and survival in a subcohort of 28 matched patients [7]. In a multivariate analysis iron chelation therapy (based on deferoxamine administration by subcutaneous infusion for at least 12 hours per day and 5 days per week) was a significant factor for overall survival (*P* = .01, hazard ratio 0,29). Similar results have been obtained also in a partially prospective analysis performed on a population of 170 MDS patients from 18 hematological centers in France. Rose et al. analyzed survival data in a cohort of MDS patients referred for transfusions in a month period in all the French centers involved. Data on survival were collected prospectively whereas transfusion and previous clinical history retrospectively. Among the cohort of iron chelated patients, 19 received deferoxamine treatment by intermittent bolus, 57 DFO by infusion for more than 3 days per week or, alternatively, DFP or DFR. The authors observed an improvement in overall survival in patients who underwent iron chelation therapy using a multivariate analysis: overall survival was 115 months in the chelated group versus 51 months in the nonchelated patients with a statistically significant difference (*P* = .0001) [8]. Although these data are promising, ad hoc prospective, controlled studies are needed in order to clarify the impact of iron chelation on overall survival and leukemic evolution in MDS.

### 7.3. Guidelines for Iron Chelation Therapy in MDS

Several international guidelines, based mostly on the opinions of expert panels, have been published in recent years, and their main features are summarized in Table 1 and reviewed by Gattermann [13]. Concerning the threshold of the number of red blood cell (RBC) transfusion for starting iron chelation, the Italian guidelines recommend chelation for patients who reached an average of 50 RBC units, but only if they have an expected lifespan of 6 months [14], while the UK expert panel considers candidates for iron chelation MDS patients who have received 25 units of blood and who will receive ongoing transfusions [15]. A similar number of previous transfusions is included in the NCCN (National Comprehensive Cancer network) guidelines, where iron chelation is suggested after the receipt of 20–30 units of RBC [16]. The Japanese guidelines considered a higher threshold of RBC units transfused; however, it should be noted that the unit in this country is smaller [17].

Most guidelines suggest as an indicator of iron overload a cut-off of serum ferritin level: even if ferritin is not such a reliable indicator of iron overload, it is easily obtained in clinical practice. The ferritin values indicated vary slighty in the guidelines (see Table 1), but the majority considered 1,000 g/L as a reasonable threshold for starting iron chelation. Serum ferritin evaluation is recommended during iron chelation to establish efficacy of the treatment at least every three months in transfusion-dependent patients. A useful and more reliable tool in monitoring iron overload and iron chelation therapy is liver magnetic resonance but it is not available in all centers. Monitoring of organ functions (mainly cardiac, hepatic and endocrine) with appropriate techniques is frequently indicated, other parameters such as ROS, NTBI and LPI are under investigation [61]. Duration of chelation has to be individually established and the therapy has to be maintioned as long as transfusion therapy continues or as long as iron overload remains clinically relevant [18].

 In summary, the majority of authors are in agreement that iron chelation should be offered to lower-risk MDS patients with a life expectancy more than one year and a serum ferritin above the mentioned threshold. Patients who are candidates for allogenic transplantation or other effective therapies can also be considered candidates for chelation.

### 7.4. Improvement in Hematopoiesis with Chelation

In some patients with MDS receiving iron chelation, improved hematopoiesis has been noted. There are some data regarding this effect with deferoxamine and deferiprone. In 1996 Jensen et al. described a hemoglobin improvement in 11 MDS patients treated with deferoxamine for up to 60 months, but in some cases there was even a trilineage response [62, 63]. It has also been reported that patients with myelofibrosis have a similar response to deferiprone [64]. In all of these reports, the hematological response was associated with a sharp decrease in iron burden and the effect was fairly long lasting, in the order of one year. The effect of deferasirox therapy seems to differ: several reports and our own experience have demonstrated an unexpected improvement in hemoglobin level and reduction in transfusion requirements even after only a few months of chelation [65–67]. Three out of five patients described in these reports became transfusion independent. A hematologic improvement by World Health Organization (WHO) criteria has also been recently described in clinical trials with deferasirox in low and int-1 MDS patients [68]. The hemopoiesis improvement has not yet been reported in any patients affected by thalassemia or other hereditary anemias, but it seems not restricted to MDS patients, as several cases of myelofibrotic patients have been observed. It is not yet clear which mechanism could lead to the hemoglobin improvement and which patients could benefit from this effect. Removing excess iron from erythroid precursors could improve erythropoiesis, similar to what occurred in a case of sideroblastic congenital anemia due to GLRX5 deficiency described recently by Camaschella et al. where an efficient iron chelation obtained by DFO subcutaneous infusion led the patients to transfusion independence due to a rebalancing in iron-responsive protein 1 and 2 functions and intracellular iron distribution [69]. Also observed was a reduced growth of erythroid colonies in vitro in iron-loaded patients: the study performed by Hartmann et al. [70] on 42 peripheral blood samples of MDS patients demonstrated a statistically significant (*P* < .004) reduced erythroid colony formation in patients with high serum ferritin levels thus supporting the hypothesis that iron chelation could induce an improvement in hemopoiesis, perhaps by reducing NTBI-mediated toxicity or, alternatively, the phenomenon could be explained by the more severity of bone marrow disease in transfusion-dependent patients. It should be kept in mind, however, that changes in hemopoiesis have been seen only in a portion of patients, while NTBI detoxification occurs in all chelated subjects according to the drug used and its type of administration. Our hypothesis to account for this discrepancy is that deferasirox could interfere directly with oncogenes in the MDS blast cell. Nuclear factor-kappaB (NF-*κ*B) is a key regulator of many cellular processes and its impaired activity has been described in different myeloid malignancies including MDS and could be a good potential target of deferasirox activity. Preliminary in vitro analysis demonstrated a deferasirox-dependent reduction in both nuclear localization and activity of NF-*κ*B in MDS cells and leukemia cell lines [71]. NF-*κ*B effects were not detected in cells incubated with DFO or DFP. The NF-*κ*B inhibition by deferasirox could prove to be an important therapeutic option in higher risk MDS patients, targeting blast cells in which increased NF-*κ*B activity has been extensively demonstrated, and acting as a possible enhancer of chemosensitivity of the neoplastic clone. Our promising hypothesis needs obviously to be validated in vivo by prospective clinical trials.

## 8. Conclusion

The usefulness and clinical indications of iron chelation therapy in myelodysplastic syndrome patients are still under debate within the scientific community [12, 72, 73]. Many authors underline the deleterious effect of iron deposition in tissues due to secondary hemochromatosis, similar to what occurs in the well-studied setting of congenital anemias. These data are mainly derived from clinical studies showing that transfusion dependence and high serum ferritin levels are independent prognostic variables for reduced overall and disease-free survival [4, 5]. High serum ferritin level was also recently identified as a prognostic factor for shorter time to progression to leukemia [9]. There are also data showing an impact of iron chelation on survival, though these data are from retrospective studies [7, 8]. The main concern of experts not in favour of iron chelation is that there is insufficient evidence about the contribution of iron overload to mortality rate and cardiac dysfunction in MDS patients, and about a positive impact of iron chelation on improving organ dysfunction, survival and quality of life. Strong prospective studies addressing these points are needed in the coming years, especially to address a putative survival effect. It is not yet clear which MDS patients could benefit most from iron chelation therapy: international guidelines are not completely in agreement on serum ferritin threshold level or number of RBC units transfused for starting iron chelation [13]. Candidates for this therapy are mainly lower-risk patients with a better prognosis and a longer life expectancy, but patients who are candidates for SCT or chemotherapy should also be included. More recently, Pullarkat proposed to change our thinking about restricting chelation to patients with lower risk MDS, considering recent data about leukemic progression and incidence of infection in iron loaded patients [12]. Finally, the data about hemopoiesis improvement and NF-*κ*B activity inhibition have the potential to provide new insight into iron chelation and its beneficial effects, all suggesting that higher risk MDS patients may also be good candidates for this therapy [66–68, 71]. In our opinion, we should change our thinking about iron chelation in MDS, expanding its indications from a mere supportive therapy for preventing organ damage due to RBC transfusions, to an active measure against progression to leukemia.

## Figures and Tables

**Table 1:** Overview of the published guidelines for iron chelation therapy in MDS patients. RBC (red blood cell), IPSS (International Prognostic Scoring System).

Guidelines	Italy 2002	UK 2003	Nagasaki Consensus 2005	Canada 2008	Spain 2008	Japan 2008	MDS Foundation 2008	Austria 2008	Israel 2008	NCCN 2009
Transfusion status	Received ≥50 RBC units	Received ~25 RBC units	Transfusion dependent	Transfusion dependent	Transfusion dependent	Received >40 Japanese units	Received 2 RBC units/month for ≥1 year	Transfusion dependent	Received 20–25 RBC units or more than 2 RBC units/month for 6 months	Received 20–30 RBC units to continue transfusions
Serum ferritin level(ng/mL)	—	—	>1000 –2000	>1000	>1000	>1000	>1000	>2000	>1000	>2500
Patients	Life-expectancy >6 months	Long-term survival likely (eg, pure sideroblastic anemia, del(5q))	Low or Int-1 IPSS Refractory anemia (RA), RA with ringed sideroblasts (RARS), del(5q)	Low or Int-1 IPSS, RA, RARS, del(5q) (Int-2 or High IPSS)	IPSS Low or Int-1, Low risk (Spanish prognostic index)	Life-expectancy >1 year	Life-expectancy >1 year	Life expectancy >2 years	Life-expectancy >1 year	Low or Int-1 IPSS
